# The Market of Biopharmaceutical Medicines: A Snapshot of a Diverse Industrial Landscape

**DOI:** 10.3389/fphar.2017.00314

**Published:** 2017-06-08

**Authors:** Evelien Moorkens, Nicolas Meuwissen, Isabelle Huys, Paul Declerck, Arnold G. Vulto, Steven Simoens

**Affiliations:** ^1^Department of Pharmaceutical and Pharmacological Sciences, KU LeuvenLeuven, Belgium; ^2^Hospital Pharmacy, Erasmus University Medical CenterRotterdam, Netherlands

**Keywords:** Off-patent biological medicine, biosimilar medicine, investment and development strategies, biopharmaceutical market, pharmaceutical industry

## Abstract

**Background:** Biopharmaceutical medicines represent a growing share of the global pharmaceutical market, and with many of these biopharmaceutical products facing loss of exclusivity rights, also biosimilars may now enter the biopharmaceutical market.

**Objectives:** This study aims to identify and document which investment and development strategies are adopted by industrial players in the global biopharmaceutical market.

**Methods:** A descriptive analysis was undertaken of the investment and development strategies of the top 25 pharmaceutical companies according to 2015 worldwide prescription drug sales. Strategies were documented by collecting data on manufacturing plans, development programs, acquisition and collaboration agreements, the portfolio and pipeline of biosimilar, originator and next-generation biopharmaceutical products. Data were extracted from publicly available sources.

**Results:** Various investment and development strategies can be identified in the global biopharmaceutical market: (a) development of originator biopharmaceuticals, (b) investment in biotechnology, (c) development of next-generation biopharmaceuticals, (d) development of biosimilars, (e) investment in emerging countries, and (f) collaboration between companies. In the top 25 pharmaceutical companies almost every company invests in originator biopharmaceuticals and in biotechnology in general, but only half of them develops next-generation biopharmaceuticals. Furthermore, only half of them invest in development of biosimilars. The companies' biosimilar pipeline is mainly focused on development of biosimilar monoclonal antibodies and to some extent on biosimilar insulins. A common strategy is collaboration between companies and investment in emerging countries.

**Conclusions:** A snapshot of investment and development strategies used by industrial players in the global biopharmaceutical market shows that all top 25 pharmaceutical companies are engaged in the biopharmaceutical market and that this industrial landscape is diverse. Companies do not focus on a single strategy, but are involved in multiple investment and development strategies. A common strategy to market biopharmaceuticals is collaboration between companies. These collaborations can as well be used to gain access in regions the company has less experience with. With patents expiring for some of the highest selling monoclonal antibodies, this snapshot highlights the interest of companies to invest in the development of these molecules and/or enter into collaborations to create access to these molecules.

## Introduction

Totaling US$ 228 billion in global sales in 2016 (Troein, 2017), biopharmaceutical medicines represent a growing share of the global pharmaceutical market. With many of these biopharmaceutical products facing loss of patent protection and other exclusivity rights, also non-innovator versions of these molecules, biosimilars, may now enter the market, resulting in a shift of market shares (IMS Health, 2016), revision of strategies of companies and attraction of new players to the biopharmaceutical market. Due to lower research and development costs and increase in competition, biosimilars offer a lower cost alternative to expensive biopharmaceutical therapies. By adopting biosimilars, health care systems can expand patient access, offer more treatment options to physicians and have a new tool to control increasing health care expenses (IMS Institute for Healthcare Informatics, 2016). Overall, large investments have been made by companies to compete on the biopharmaceutical market.

These developments are also reflected in the industrial players in this market. Although there does not exist a classification system of companies active in this market, one could distinguish between big pharmaceutical companies, biotechnological companies, generics companies, new entrants, and companies from emerging countries. Big pharmaceutical companies are companies like Pfizer, Merck, and J&J, which originally focused on chemically developed medicines, and now target the biopharmaceutical market. On the biopharmaceutical market, there are as well biotechnological companies, like Amgen, whose focus has been on the development of biopharmaceutical medicines, be it initially originator medicines and in a later phase biosimilar medicines. Generics companies, companies originally focusing on generics, have also entered the biosimilar market (e.g., Sandoz). New entrants are new biotechnological companies, such as Celltrion and Samsung Bioepis. Companies from emerging countries, like Biocon and Dr. Reddy's, are companies in fast-developing economies.

The aim of this original research article is to identify and document which investment and development strategies are adopted by industrial players in the global biopharmaceutical market. To this effect, we distinguish between various investment and development strategies, and exemplify these strategies for the top 25 pharmaceutical companies. In 2012, Calo-Fernández et al. identified different players active in the biosimilar industry and core capabilities to enter this market, supported by three case studies (Calo-Fernández and Martínez-Hurtado, 2012). To the best of the authors' knowledge, our study is the first to provide a comprehensive snapshot of the industrial landscape of the biopharmaceutical and biosimilar market as of December 2016. It should be realized however that the landscape is rapidly evolving.

## Methods

A descriptive analysis was undertaken of the investment and development strategies of the top 25 pharmaceutical companies according to 2015 worldwide prescription drug sales (Evaluate Pharma, 2016; Pharm Exec, 2016). Identification of various investment and development strategies was based on previous research (Meuwissen, 2016). Identified strategies were further documented by collecting data on manufacturing plans, development programs, acquisition and collaboration agreements, the portfolio and pipeline of biosimilar, originator and next-generation biopharmaceutical products of these companies. Data were extracted from multiple, publicly available sources, including a review of the literature in PubMed and Embase over the last 5 years up to March 2016, a search of the reference list of included articles for other relevant studies, articles known to the authors, the website of the Generics and Biosimilars Initiative (GaBI) journal, GaBI Online, company and news websites. A detailed list with references consulted for each company is available from the authors on request.

## Results

Based on our analysis of industrial players in the global biopharmaceutical market, we distinguished between the following investment and development strategies: (a) development of originator biopharmaceuticals, (b) investment in biotechnology, (c) development of next-generation biopharmaceuticals, (d) development of biosimilars, (e) investment in emerging countries, and (f) collaboration between companies. Table 1 shows the investment and development strategies of the top 25 pharmaceutical companies. It shows whether the company is an originator company, whether they invest in biotechnology via investment in their own development program, via acquisition of biotechnological companies, or both. The table also shows involvement in development of next-generation biopharmaceuticals. A next-generation biopharmaceutical is created by modifying the structure of an existing biological molecule (via e.g., pegylation, glycosylation) to alter pharmacokinetic or pharmacological properties, such as half-life or bioavailability or to improve its safety profile e.g., by reducing immunogenicity. This definition of next-generation biopharmaceuticals does not include new dosage forms. Subsequently, the table shows involvement in biosimilar development. Importantly, the term biosimilar is only applicable when strict regulatory requirements (European Medicines Agency (EMA), Food and Drug Administration (FDA) guidelines) are in place in the region in which it has been approved. Table 1 also shows, the presence and investment of the company in emerging countries (focus on BRIC-countries: Brazil, Russia, India, and China). The last column provides information on collaborations between companies, this includes also co-marketing of products. Categories are not mutually exclusive, for example, next-generation biopharmaceuticals can also be classified as originator biopharmaceuticals. Furthermore, examples provided in Table 1 are not exhaustive.

**Table 1:** Top 25 pharmaceutical companies ranked by 2015 worldwide prescription drug sales and examples of their investment and development strategies in the global biopharmaceutical market as of December 2016.

**Rank (6, 7)**	**Company**	**Strategy biopharmaceutical market**						
		**(a) Development of originator biopharmaceuticals**	**(b) Investment in biotechnology**		**(c) Development of next-generation biopharmaceuticals**	**(d) Development of biosimilars**	**(e) Investment in emerging countries**	**(f) Collaboration and co-marketing**
			**Own development program**	**Acquisition**				
1	Pfizer	° Genotropin®/Genotonorm®-somatropin ° Prevnar 13®	° Investment in Global Biotechnology Center China ° Investment in new biologics clinical manufacturing facility (expansion existing site) in Andover (US) ° Plans expansion biologics plant Dublin (Ireland) ° Expansion plant Adelaide (Australia) to produce biosimilar pegfilgrastim	° Medivation (2016) ° Hospira (2015) ° Wyeth (2009)	° Development of next-generation human growth hormone in collaboration with OPKO	° Via Hospira: ° Epoetin zeta (Retacrit®) ° Filgrastim (Nivestim®) ° Infliximab (Inflectra®) ° Pipeline: adalimumab, bevacizumab, infliximab (outside EEA), rituximab, trastuzumab, pegfilgrastim (via Hospira) biosimilars	° Production facilities and sale China (biosimilars), Russia	° Celltrion (biosimilar infliximab) ° Merck KGaA (avelumab) ° OPKO (long-acting human growth hormone)
2	Novartis	° Lucentis®-ranibizumab ° Xolair®-omalizumab ° Simulect®-basiliximab ° Cosentyx®-secukinumab	° New biologics production plant Singapore ° Expansion Center of Biotechnology Huningue (France) ° Investment in biologics manufacturing sites Schaftenau and Kundel (Austria)	° Admune Therapeutics (2015)	– ° Albinterferon alfa-2b (Joulferon®) (withdrawn from market)	° Via Sandoz, the generics and biosimilars division of Novartis: ° somatropin (Omnitrope®) ° epoetin alfa (Binocrit®) ° filgrastim (Zarzio®/Zarxio®) ° etanercept (Erelzi®) ° Via subsidiary Hexal: ° epoetin alfa (Epoetin alfa Hexal®) ° filgrastim (Filgrastim Hexal®) ° Pipeline: infliximab (Sandoz acquired development and commercialization rights for EEA from Pfizer), rituximab, adalimumab, pegfilgrastim biosimilars	° Expanding presence in emerging markets of Asia, Africa and Latin America	° Genentech (development of Lucentis® and Xolair®) ° Xencor (bispecific antibodies)
3	Roche	° MabThera®/Rituxan®-rituximab ° Avastin®-bevacizumab ° Herceptin®-trastuzumab ° RoActemra®-tocilizumab ° Perjeta®-pertuzumab ° Gazyva®-obinutuzumab	° Investment in increased manufacturing capacity at sites in Vacaville (Genentech) and Oceanside (US), and Penzberg (Germany) ° Construction antibody-drug conjugate manufacturing facility Basel (Switzerland)	° Adheron Therapeutics (2015) ° InterMune (2014) ° Genentech (2009)	° Methoxy polyethylene glycol-epoetin beta (Mircera®) ° Peginterferon alfa-2a (Pegasys®)	–	° China for, amongst others, oncology treatments ° The ‘Blue Tree’ cancer patient support initiative India ° Roche Pharma Africa Strategy to improve access to treatment in Sub-Saharan Africa, focus on hepatitis and cancer in women	° Chugai Pharmaceutical (Japan) ° Pieris Pharmaceuticals (Cancer immunotherapy)
4	Merck US (MSD)	° Keytruda®-pembrolizumab ° IntronA®-interferon alfa-2b ° Several vaccines	° Investment vaccine manufacturing site Carlow (Ireland) to produce oncology biologics (Keytruda®) ° New biologics facility at site in Cork (Ireland) ° New manufacturing facility in Hangzhou (China)	° cCAM Biotherapeutics (2015)	° Peginterferon alfa-2b (PegIntron®) ° Corifollitropin alfa (Elonva®)	° Pipeline: Via collaboration Samsung Bioepis, e.g., insulin glargine, adalimumab, trastuzumab biosimilars	° Undertaking opportunities to expand in emerging markets (China, India, Brazil, Russia)	° Samsung Bioepis (development of biosimilars, commercialization of infliximab biosimilar [worldwide ex-EU/Russia/Turkey] and etanercept biosimilar [worldwide ex-US/EU/Japan]) ° J&J (commercialization Remicade®; Simponi® via Schering-Plough) ° Ablynx (nanobodies) ° Hanwha Chemical (development and commercialization etanercept biosimilar)
5	Sanofi	° Lovenox®-enoxaparin ° Lemtrada®-alemtuzumab ° Zaltrap®-ziv-aflibercept ° Vaccines (via Sanofi-Pasteur)	° Expansion biologics site Geel (Genzyme, Belgium) to manufacture mAbs ° Investment Genzyme production facility Framingham (US)	° Shantha Biotechnics (2013) ° Genzyme (2011)	° Insulin glargine-Lantus® ° insulin glulisine -Apidra®	° Pipeline: insulin lispro biosimilar	° Focus on China ° Via acquisition of Shantha Biotechnics (focus vaccines) ° Increasing insulin production in Russia	° Regeneron (Zaltrap®-aflibercept)
6	Gilead Sciences	° Macugen®-pegaptanib ° Lexiscan®-regadenoson	° Acquisition of biologics manufacturing plant in Oceanside (US) from Genentech	° Arresto Biosciences (2011)	–	–	– ° No specific efforts for biopharmaceuticals, focus on HIV treatments	– ° Not for biopharmaceuticals, focus on HIV treatments
7	Johnson & Johnson	° Remicade®-infliximab ° Eprex®/Erypo®-epoetin alfa ° Simponi®-golimumab ° Stelara®-ustekinumab	° Biologics manufacturing site Cork (Ireland) ° Biologics manufacturing site Leiden (The Netherlands)	° Crucell (2010) ° Centocor (1999)	–	–	° Global policy to support access in emerging markets as well	° MSD (Remicade®, Simponi®) ° Genmab (bispecific antibodies) ° Bavarian Nordic (Ebola vaccine)
8	GlaxoSmithKline	° Benlysta®-belimumab ° Bexxar®-tositumomab ° Multiple vaccins	° Investment vaccines manufacturing site Tuas (Singapore) ° Investments in UK manufacturing network ° New US vaccines R&D center	° GlycoVaxyn (2015) ° Human Genome Sciences (2012) ° CellZome (2011)	° Albiglutide (Eperzan®/Tanzeum®)	–	° Increasing investment in emerging markets, biggest areas of growth	° Genmab (Arzerra®- ofatumumab; rights now transferred to Novartis) ° OncoMed (development oncology drugs)
9	AstraZeneca	° Synagis®-palivizumab ° Vaccines	° Construction biologics plant Södertälje (Sweden) ° Investment biologics production facility Frederick (US) ° Acquisition commercial biologics manufacturing site Boulder and supporting warehouse Longmont (US)	° Spirogen (2013) ° MedImmune (2007) ° Cambridge Antibody Technology (2006)	° Via biologics arm: MedImmune	° Pipeline: rituximab, bevacizumab biosimilars	° Area of focus, specifically China	° Samsung BioLogics (joint venture: Archigen Biotech, development rituximab biosimilar) ° Fujifilm Kyowa Kirin Biologics (development bevacizumab biosimilar) ° MSD (Manufacturing capacity sharing) ° Celgene (durvalumab development) ° Eli Lilly (immuno-oncology drugs) ° Moderna Therapeutics (immuno-oncology mRNA therapeutics) ° Regeneron (antibody-drug conjugates) ° Inovio (cancer vaccines)
10	AbbVie	° Humira®-adalimumab ° Synagis®-palivizumab	° New manufacturing facility Tuas (Singapore)	° Stemcentrx (2016) ° Pharmacyclics (2015)	–	–	° AbbVie will focus on expanding presence in emerging markets ° Via manufacturing plant Singapore	° Boehringer Ingelheim (immunology drugs) ° Bristol-Myers Squibb (oncology)
11	Amgen	° Blincyto®-blinatumomab ° Enbrel®-etanercept ° Epogen®-epoetin alfa ° Neupogen®-filgrastim ° Prolia®/Xgeva®-denosumab ° Repatha®-evolocumab ° Vectibix®-panitumumab	° Several production facilities focused on biologics ° New manufacturing facility Tuas (Singapore) ° Several biopharmaceutical medicines in pipeline (e.g., monoclonal/bispecific antibodies, fusion proteins)	° Micromet (2012) ° BioVex Group (2011)	° Darbepoetin alfa (Aranesp®) ° pegfilgrastim (Neulasta®)	° Adalimumab (Amjevita®) ° Pipeline: trastuzumab, bevacizumab, infliximab, rituximab, cetuximab	° Plans for increased presence in key new and emerging markets (e.g., China, Latin America, Middel East; via collaborations) ° Via Dr Reddy's (India)	° Allergan/Actavis (biosimilar monoclonal antibodies for oncology) ° Daiichi Sankyo (biosimilars) ° Dr Reddy's (commercialization Xgeva®, Vectibix® and Prolia® in India) ° UCB (romosozumab) ° Advaxis (cancer immunotherapy) ° Xencor (cancer immunotherapy and inflammation)
12	Allergan	° Botox®-onabotulinumtoxinA	° Biosimilar development center Liverpool (UK)	° RetroSense Therapeutics (2016) ° Motus Therapeutics (2016)	–	° Pipeline: Biosimilar program for oncology (Amgen)	° Presence in all continents of the world ° Actively investing in South Korea, China, Poland, Turkey, Philippines, South Africa, Russia, Indonesia and Vietnam	° Amgen (biosimilar monoclonal antibodies for oncology) ° Molecular Partners (DARPin-based products)
13	Teva Pharmaceutical Industries	° Cinqair®-reslizumab ° Granix®- tbo-filgrastim	° Investment biotechnological production site Ulm (Germany)	° Labrys biologics (2014) ° CoGenesys (2008)	° Lipegfilgrastim (Lonquex®) ° Development of next-generation human growth hormone	° Filgrastim (Tevagrastim®) ° follitropin alfa (Ovaleap®) ° filgrastim (Ratiograstim®) via acquisition Ratiopharm	° Via Teva Growth Markets	° Celltrion (biosimilars, e.g., trastuzumab, rituximab commercialization in US and Canada) ° Regeneron (fasinumab)
14	Novo Nordisk	° Several insulins ° Norditropin®-somatropin ° Novoseven®-recombinant factor VIIa ° NovoEight®-recombinant factor VIII ° Novo Thirteen®-recombinant factor XIII	° Expansion production facilities US and Denmark for diabetes portfolio and hemophilia treatments	° Calibrium (2015) ° MB2 (2015)	° Liraglutide (Saxenda®/Victoza®) ° Insulin detemir (Levemir®) ° Insulin degludec (Tresiba®) ° Development of semaglutide (GLP-1 analog) ° Development of next-generation human growth hormone	–	° Core capability: building and maintaining a leading position in emerging markets ° Investments in China and Russia for R&D, production and sales ° Changing Diabetes for Children program, training HCP in poorest countries in the world	° Ablynx (nanobodies) ° Xencor (bispecific antibodies)
15	Eli Lilly	° Forteo®-teriparatide ° Glucagon®-glucagon ° Humulin®-insulin ° Humatrope®-somatropin ° Taltz®-ixekizumab ° Cyramza®-ramucirumab ° Lartruvo®-olaratumab ° Portrazza®-necitumumab	° Construction of commercial scale biologics facility Cork (Ireland) ° Expansion Lilly Biotechnology Center San Diego (US) ° Continuous investment in insulin producing sites	° SGX Pharmaceuticals (2008) ° ImClone Systems (2008) ° Icos Corporation (2007)	° Insulin lispro (Humalog®) ° Dulaglutide (Trulicity®)	° Insulin glargine (Abasaglar®/Basaglar®)	° Emerging markets business area ° China via Yabao Pharmaceuticals for diabetes ° China via Innovent Biologics	° Boehringer Ingelheim (Abasaglar®) ° AstraZeneca (MedImmune; immune-oncology drugs) ° Pfizer (tanezumab, chronic pain) ° Merck KGaA (manufacturing and commercialization cetuximab US and Canada)
16	Bayer	° Eylea®-aflibercept ° Betaferon®-interferon beta-1b ° Kogenate®-octocog alfa	° Expansion sites Germany and US for hemophilia-A products	° DIREVO Biotech (2008) ° Schering (2006)	° Development damoctocog alfa pegol (pegylated octocog alfa)	–	° Increasing sales in emerging markets	° Regeneron (aflibercept for eye diseases) ° OncoMed (development oncology drugs) ° Compugen (development and marketing antibody-based cancer therapeutics)
17	Bristol-Myers Squibb	° Orencia®-abatacept ° Opdivo®-nivolumab ° Yervoy®-ipilimumab ° Empliciti®-elotuzumab ° Nulojix®-belatacept	° Expansion biologics manufacturing facility Devens, Massachusetts (US) ° Construction of new large-scale manufacturing facility in Cruiserath (Ireland) ° Establishment of biomanufacturing process laboratory Dublin (Ireland)	° Padlock therapeutics (2016) ° Cormorant Pharmaceuticals (2016) ° iPierian (2014) ° ZymoGenetics (2010) ° Medarex (2009) ° Adnexus Therapeutics (2007)	° Development of pegylated fibroblast growth factor 21	–	° Expanding capacity in China ° Partnership agreements in China and Singapore, e.g., Simcere Pharmaceutical Group for abatacept ° R&D center in Bangalore (India)	° AbbVie (oncology) ° Samsung BioLogics (Production biopharmaceuticals at South-Korea plant) ° Janssen (immuno-oncology)
18	Takeda	° Entyvio®-vedolizumab ° Adcetris®-brentuximab vedotin	° Acquisition biologics manufacturing facility Minnesota (US) ° Construction vaccine manufacturing plant Singen (Germany)	° Inviragen (2013) ° Multilab (2012) ° LigoCyte (2012) ° Millenium Pharmaceuticals (2008)	–	–	° Enhancing position in Brazil via acquisition Multilab (2012) ° Emerging markets business unit headquartered in Biopolis (Singapore)	° Amgen (Marketing Japan e.g., panitumumab, etanercept) ° Crescendo Biologics (Humabody®-based therapeutics) ° MacroGenics (bispecific antibodies)
19	Boehringer Ingelheim	° Praxbind®-idarucizumab	° Contract manufacturing business via BioXcellence ° Large scale facilities in Biberach (Germany, contract manufacturing) ° Investments plant Vienna (Austria, contract manufacturing) ° Acquisition biologics plant Fremont (US, contract manufacturing)	–	–	° Insulin glargine (Abasaglar®/Basaglar®) ° Pipeline: bevacizumab, adalimumab biosimilars	° Biologics contract manufacturing site Shanghai (China)	° Eli Lilly (Abasaglar®) ° Xencor (monoclonal antibodies) ° Zealand (therapeutic proteins diabetes/obesity)
20	Astellas Pharma	° Eligard®-leuprolide acetate ° Amevive®-alefacept	–	° Ocata therapeutics (2016) ° Ganymed Pharmaceuticals (2016)	–	–	° Reinforce sales platform, focus on China and Russia	° Vical (cytomegalovirus vaccin) ° Amgen (joint venture Amgen Astellas Biopharma, development and co-marketing Japan) ° Seatle genetics (Enfortumab Vedotin)
21	Mylan	–	–	–	–	° Pipeline: Collaboration Biocon trastuzumab, adalimumab, bevacizumab, pegfilgrastim biosimilars, insulin analogs; collaboration Momenta abatacept biosimilar; collaboration Mabion rituximab biosimilar	° Via Biocon for biosimilars	° Biocon (biosimilars e.g., trastuzumab, pegfilgrastim, adalimumab, bevacizumab; insulin analogs) ° Momenta (biosimilars e.g., abatacept) ° Mabion (commercial rights rituximab biosimilar candidate)
22	Biogen	° Avonex®-interferon beta-1a ° Tysabri®-natalizumab ° Zinbryta®-daclizumab	° New biologics manufacturing facility Luterbach (Switzerland)	° Stromedix (2012)	° Efmoroctocog alfa (Eloctate®/Elocta®) ° Eftrenonacog alfa (Alprolix®) ° Peginterferon beta-1a (Plegridy®)	° Via Samsung bioepis: ° etanercept (Benepali®) ° Infliximab (Flixabi®) ° Pipeline: Via Samsung Bioepis adalimumab, insulin glargine, trastuzumab, bevacizumab biosimilars	° Partnering with UCB for marketing products in emerging markets ° Via Samsung Bioepis for biosimilars	° Samsung BioLogics (via Samsung Bioepis, development biosimilars) ° Roche (co-development and marketing Rituxan® US, Gazyva® US) ° AbbVie (co-promotion Zinbryta® in US) ° Sobi (Alprolix®, Eloctate®) ° MSD (via Samsung Bioepis for commercialisation Benepali® and Flixabi®)
23	Celgene	–	° Looking to hire talented people for biologics development	° EngMab (2016) ° Abraxis BioScience (2010)	–	–	– ° Not for biopharmaceuticals	° OncoMed (development oncology drugs) ° AstraZeneca (MedImmune: durvalumab, oncology) ° Sutro Biopharma (multispecific antibodies, antibody-drug conjugates) ° Northern Biologics (antibodies cancer and fibrosis) ° Acceleron (protein therapeutics cancer and orphan diseases)
24	Merck KGaA	° Erbitux®-cetuximab ° Rebif®-interferon beta-1a ° GONAL-f® – follitropin alfa ° Pergoveris®-follitropin alfa/lutropin alfa ° Luveris®-lutropin alfa ° Ovitrelle®-choriogonadotropin alfa ° Cetrotide®-cetrorelix ° Saizen®-somatropin	° Merck Biotech Center Corsier-sur-Vevey (Switzerland) ° Investment in Biosimilars Unit	° BioReliance (2012) ° Molecular Medicine BioServices (2007) ° Serono (2006) ° Biochrom AG (2012) ° CellASIC (2012)	–	° Pipeline: E.g., bevacizumab, cetuximab, etanercept, infliximab, rituximab, adalimumab, and trastuzumab biosimilars	° R&D and manufacturing facilities in China to provide Chinese market ° Via partnership with Indian firm Lupin ° Plans to expand presence in Africa	° Dr. Reddy's (biosimilars oncology) ° Bionovis (biosimilars) ° Pfizer (avelumab) ° MorphoSys (antibodies) ° Sutro (antibody drug conjugates) ° Mersana (antibody drug conjugates)
25	Daiichi Sankyo	° Vaccines Japan	° Vaccine business unit	° U3 Pharma (2008)	–	° Pipeline: Collaboration Amgen e.g., adalimumab, bevacizumab, trastuzumab	– ° Not for biopharmaceuticals	° AstraZeneca (commercialization FluMist® Japan) ° Amgen (biosimilars Japan) ° CMC Biologics (antibodies)

Examples: non-exhaustive list

EEA, European Economic Area; EU, European Union; J&J, Johnson&Johnson; mAb, monoclonal antibody, R&D, Research and Development; US, United States of America

The first column of Table 1 shows that 23 of the 25 companies listed (except for Mylan and Celgene) have originator biopharmaceuticals. However, Celgene has several originator biopharmaceuticals under development. The top 20 companies all have originator biopharmaceuticals on the market.

Twenty-three out of 25 companies invest in biotechnology by investing in their own development program and infrastructure. Also 23 companies invest in biotechnology via acquisition of biotechnological companies. Mylan did no acquisitions or investments in its own infrastructure, its presence on the biopharmaceutical market is limited to collaborations for biosimilar development. Mylan is the only company in the list that only engages in biosimilar development and is not focusing on originator biopharmaceuticals/innovation. The top 10 companies all have originator products and invest in biotechnology via investment in their own development program as well as via acquisitions.

Almost every company in the top 25 invests in originator biopharmaceuticals and in biotechnology in general, but only half of them develops next-generation biopharmaceuticals. Furthermore, only half of them invest in development and marketing of biosimilars. Six companies [6, 7, 10, 18, 20, 23] (numbers between square brackets indicate the position of the company in Table 1) only have originator biopharmaceuticals, and no next-generation biopharmaceuticals or biosimilars. Eight companies [1, 4, 5, 9, 11, 13, 15, 22] invest in next-generation biopharmaceuticals and also in biosimilars. Five companies [3, 8, 14, 16, 17] only invest in next-generation biopharmaceuticals and not in biosimilars, six companies [2, 12, 19, 21, 24, 25] invest in biosimilars, but not in next-generation biopharmaceuticals. These next-generation biopharmaceuticals are often a modified version of the companies' own originator biopharmaceuticals. A distinction can be made between biosimilars of less complex molecules (insulin, follitropin, epoetin, filgrastim, somatropin) and biosimilars of monoclonal antibodies (mAbs). Four companies [5, 13, 15, 19] only invest in biosimilars of less complex molecules, five companies [9, 11, 12, 24, 25] only in biosimilars of monoclonal antibodies, and five companies [1, 2, 4, 21, 22] invest in both. When looking at the pipeline of the companies, the focus of biosimilar development is mainly on biosimilar monoclonal antibodies and to some extent on biosimilar insulins. For example, Sanofi is making a biosimilar version of insulin lispro, a product of competitor Eli Lilly, whereas Eli Lilly has a biosimilar of Sanofi's insulin glargine.

Twenty-two out of 25 companies are actively expanding their presence in emerging markets. These are companies which already have biopharmaceutical products on the market and are focused on biopharmaceuticals. Gilead's focus is not on biopharmaceuticals. Celgene and Daiichi Sankyo do not market biopharmaceuticals yet.

Collaboration between companies is a common strategy for developing and marketing biopharmaceuticals. All but one company in the list, Gilead [6], are collaborating with other companies or are engaged in co-marketing. Seven companies use all six investment and development strategies [1, 4, 5, 11, 13, 15, 22].

## Discussion

As shown in the study by Calo-Fernández and Martínez-Hurtado (2012), in the 1990s big pharmaceutical companies and generics companies developed an interest in the market segment of biotechnology, and innovative biotechnological companies saw the potential of biosimilars. We again looked at the evolving industrial landscape, but now with biosimilars being an established option for all type of companies.

This article has identified the following investment and development strategies used by industrial players in the global biopharmaceutical market: (a) development of originator biopharmaceuticals, (b) investment in biotechnology, (c) development of next-generation biopharmaceuticals, (d) development of biosimilars, (e) investment in emerging countries, and (f) collaboration between companies. Each of these six investment and development strategies is discussed in the following paragraphs.

### Development of originator biopharmaceuticals

Companies can look for new possibilities in diagnosis, prevention and treatment of chronic diseases, cancer,… and choose to develop originator biopharmaceuticals. Companies investing in originator biopharmaceuticals will, thanks to their investment in research and development (R&D) and new products, have a competitive advantage over companies mainly focusing on development of biosimilars, as they are able to charge a premium price for their originator molecules. However, the risk exists that the market share of the reference product declines due to competition with biosimilars and other non-originator products. If new originator molecules are not ready to follow up, revenue is lost. Big pharmaceutical companies that solely invest in innovation are companies like Roche, GSK, AbbVie, and J&J. AbbVie owns with Humira® one of the highest selling medicines in the world, with global sales in 2016 of US$ 16.1 billion (AbbVie, 2017). While facing loss of exclusivity rights in US in December 2016 and in Europe in 2018 (GaBI Online-Generics and Biosimilars Initiative, 2015), a key challenge will be to retain market share with new biopharmaceutical products. Until now, the answer is often a new formulation with a different concentration, which cannot be copied by biosimilar developers. Roche, which invested in several originator monoclonal antibodies (e.g., trastuzumab, rituximab, bevacizumab, pertuzumab), is just faced with competition from biosimilars with the first rituximab biosimilar being licensed in the EU, although patent and exclusivity rights of several molecules expired years ago (rituximab, 2013; trastuzumab, 2014) (GaBI Online-Generics and Biosimilars Initiative, 2015; F. Hoffmann-La Roche Ltd., 2016). Roche, as a developer of complex molecules keeps focusing on innovation (Roche, 2014). The company developed subcutaneous forms of its intravenous medicines rituximab and trastuzumab in order to increase patients' convenience (Roche, 2016).

### Investment in biotechnology

When companies not established as biotechnological companies (e.g., traditional, big pharmaceutical companies) wish to enter the biopharmaceutical market, they would need the right infrastructure and knowledge. This can be achieved via acquisition of biotechnological companies. In this way, they can link their image and marketing to the experience and knowledge biotechnological companies have in developing biopharmaceutical medicines. For instance, the acquisition of Genentech by Roche, or MedImmune by AstraZeneca. When companies do not invest in their own development program or infrastructure, this can mean the company works via contract manufacturers. Boehringer Ingelheim, as a contract manufacturer, did no acquisitions of biotechnological companies, but is now collaborating with several companies on the development of biosimilars.

### Development of next-generation biopharmaceuticals

As a company focusing on innovation, the development of next-generation product Mircera®, a long-acting epoetin, fits in Roche's business strategy. These next-generation biopharmaceuticals are often characterized by higher bioavailability, increased half-life, lower immunogenicity…to create added value over existing products. Health care payers and hospitals will have to assess whether these products are cost-effective. Also Amgen, as one of the first manufacturers of biopharmaceutical medicines, remains an important player due to their investment in next-generation biopharmaceuticals. With Aranesp® (darbepoetin) and Neulasta® (pegfilgrastim), two products with an increased half-life relative to the originator, Amgen can keep market shares high in the G-CSF and epoetin market. By December 2016, four biosimilars to pegfilgrastim were under evaluation at the EMA (Amgen, 2016; EMA, 2016).

### Development of biosimilars

Companies can choose to focus on development of biosimilars, like Hospira (acquired by Pfizer in 2015) and Sandoz did. Sandoz, which was first to launch a biosimilar in a highly regulated market (Europe), has the highest market share of the biosimilar market (figures up to 2014) (Long, 2015), with biosimilars of somatropin, epoetin, filgrastim, and etanercept. Table 1 shows that the focus of current biosimilar development is mainly on biosimilar monoclonal antibodies, and insulins. This market is not yet as developed as the biosimilar market with growth hormone, filgrastim and epoetin. Although many companies want a share of the biosimilar market, Merck KGaA is exploring to sell its biosimilar business (O'Donnell and Roumeliotis, 2016). Merck US has attempted to enter the biologics market via a biosimilar pathway, and recently entered into licensing agreements with e.g., Samsung Bioepis (Table 1). Similarly, Biogen, one of the first originator biotechnology companies, is now combining their expertise with biosimilars via a joint venture with Samsung BioLogics, Samsung Bioepis. Pfizer was one of the first innovator companies to set up a broad biosimilar development program (Nguyen, 2012). In addition to this, Pfizer took over Hospira for, amongst other things, its biosimilar portfolio.

### Investment in emerging countries

Emerging countries can be attractive for companies to invest in, a large market is available to supply and economic growth is expected to rise rapidly. Companies can enter emerging countries via collaborations with local manufacturers. Mylan, for example, is working with Biocon, an Indian biotechnology company, to develop biosimilar monoclonal antibodies. Companies can also enter emerging countries by opening their own facilities or by starting initiatives to provide access to treatment in these markets (e.g., Roche's patient support initiative, the “Blue Tree,” for cancer care in India). Overall, all companies in the top 25 invest in emerging countries. Some just not yet for biopharmaceutical medicines.

### Collaboration between companies

Collaboration between companies has been a common strategy for marketing pharmaceuticals for decennia, and is also used for biopharmaceuticals. In this way, the combined experience of companies can be used in synergy to compete on the market. Companies can work together with biotechnological companies from emerging countries in order to obtain a place on the local market. For smaller biotechnological companies, collaboration with a large, reputable pharmaceutical company can help to increase trust in their product by physicians and patients. The knowhow delivered by biotechnological companies may, in combination with a well-defined market strategy of big pharmaceutical companies, aid in enhancing the uptake of a new product. New entrants and companies from emerging countries may increase trust by collaborations with established companies in the biopharmaceutical market. Another factor that may play a role in entering into an agreement between companies is risk sharing, where profits and losses are shared between companies. The development cost of a biopharmaceutical medicine is traditionally higher than that of a chemically developed medicine, consequently failure to develop and market a biopharmaceutical medicine may have serious financial implications. Examples of this strategy with respect to biosimilars are the arrangements made by new market entrants, such as Celltrion and Samsung Bioepis, with more established companies. Celltrion is collaborating with Hospira (Pfizer) in different regions in the world, and with Mundipharma and Orion in Europe. Likewise, Samsung Bioepis has a co-investment strategy with US biotech originator company Biogen, and with Merck US.

### General aspects

It is interesting to note that in the top 10 five companies (50%) and in the top 25 eleven companies (44%) currently have not entered in the development or marketing of biosimilars. It suggests that companies deliberately choose whether or not to enter the biosimilar market.

This study is subject to a number of limitations. The analysis is limited to the top 25 pharmaceutical companies, and new entrants like Samsung BioLogics are not (yet?) in the top 25, although it can be argued that mostly big companies have the resources, capacity and expertise needed to invest in biopharmaceutical medicines. This article only gives a static snapshot anno 2016 of a dynamic industrial landscape and follow-up is needed to investigate changes in the global biopharmaceutical market. In this respect, it should be noted that now that patents of new classes of biopharmaceutical products (e.g., mAbs in oncology) expire, many companies revise their strategy. For instance, Amgen will, as an innovator with originator medicines and next-generation products, focus on the development of biosimilars of monoclonal antibodies. Amgen will use its experience as an innovator to compete with other biosimilar developers. Table 1 only provides a qualitative overview of the investment and development strategies used by different players in the biopharmaceutical market and does not give quantitative information like sales figures and amounts invested. Furthermore, only publicly available information was consulted, as access to inside business information, often confidential, was missing. It can be noted that there is a lack of peer-reviewed scientific articles providing data on investment and development strategies, and therefore extensive use has been made of gray literature. The classification system is not specific to the biopharmaceutical market. However, its broad application can be valuable in further research and analysis of the market of other types of medicines. To the best of the authors' knowledge, this is the first study to provide a systematic overview of investment and development strategies adopted by industrial players in the global biopharmaceutical market.

## Conclusion

This article presented a snapshot of investment and development strategies used by industrial players in the global biopharmaceutical market. This snapshot shows that all top 25 pharmaceutical companies are engaged in the biopharmaceutical market and that this industrial landscape is diverse. Companies can develop biosimilars or can decide to solely focus on innovation, can seek support from biotechnological companies, or target emerging countries. Companies do not focus on a single strategy, but are involved in multiple investment and development strategies. A common strategy to market biopharmaceutical medicines is collaboration between companies, whether or not from different regions in the world. These collaborations can as well be used to gain access in regions the company has less experience with. With patents expiring for some of the highest selling monoclonal antibodies, this snapshot highlights the interest of companies to invest in the development of these molecules and/or enter into collaborations to create access to these molecules.

## Author contributions

SS, IH, AV, EM, and NM developed the idea for and were involved in the design of this study. EM and NM reviewed available data sources and drafted the initial version of the manuscript. IH, AV, PD, and SS critically revised the manuscript. All authors read and approved the final manuscript.

### Conflict of interest statement

SS, IH, PD, and AV are the founders of the KU Leuven Fund on Market Analysis of Biologics and Biosimilars following Loss of Exclusivity. SS, IH, and AV are conducting biosimilar research sponsored by Hospira (now Pfizer). SS is involved in a stakeholder roundtable on biosimilars sponsored by Amgen, Pfizer and MSD, and has participated in an advisory board meeting on biosimilars for Pfizer. AV is involved in consulting, advisory work and speaking engagements for a number of companies, a.o. AbbVie, Amgen, Biogen, EGA, Pfizer/Hospira, Mundipharma, Roche, Sandoz. PD participated at advisory board meetings for AbbVie, Amgen and Hospira, and is on the Speakers' Bureau of AbbVie, Celltrion, Hospira, Merck Serono, and Roche. EM and NM declare that the research was conducted in the absence of any commercial or financial relationships that could be construed as a potential conflict of interest.
